# Supplementary material for: An Inducible ESCRT-III Inhibition Tool to Control HIV-1 Budding
Source: Viruses. 2023 Nov 22;15(12):2289. doi: 10.3390/v15122289 (PMC10748027; doi:10.3390/v15122289)
Supplement: Supplementary file 1 [file viruses-15-02289-s001.zip › Figure S1.pptx]

## Slide 1
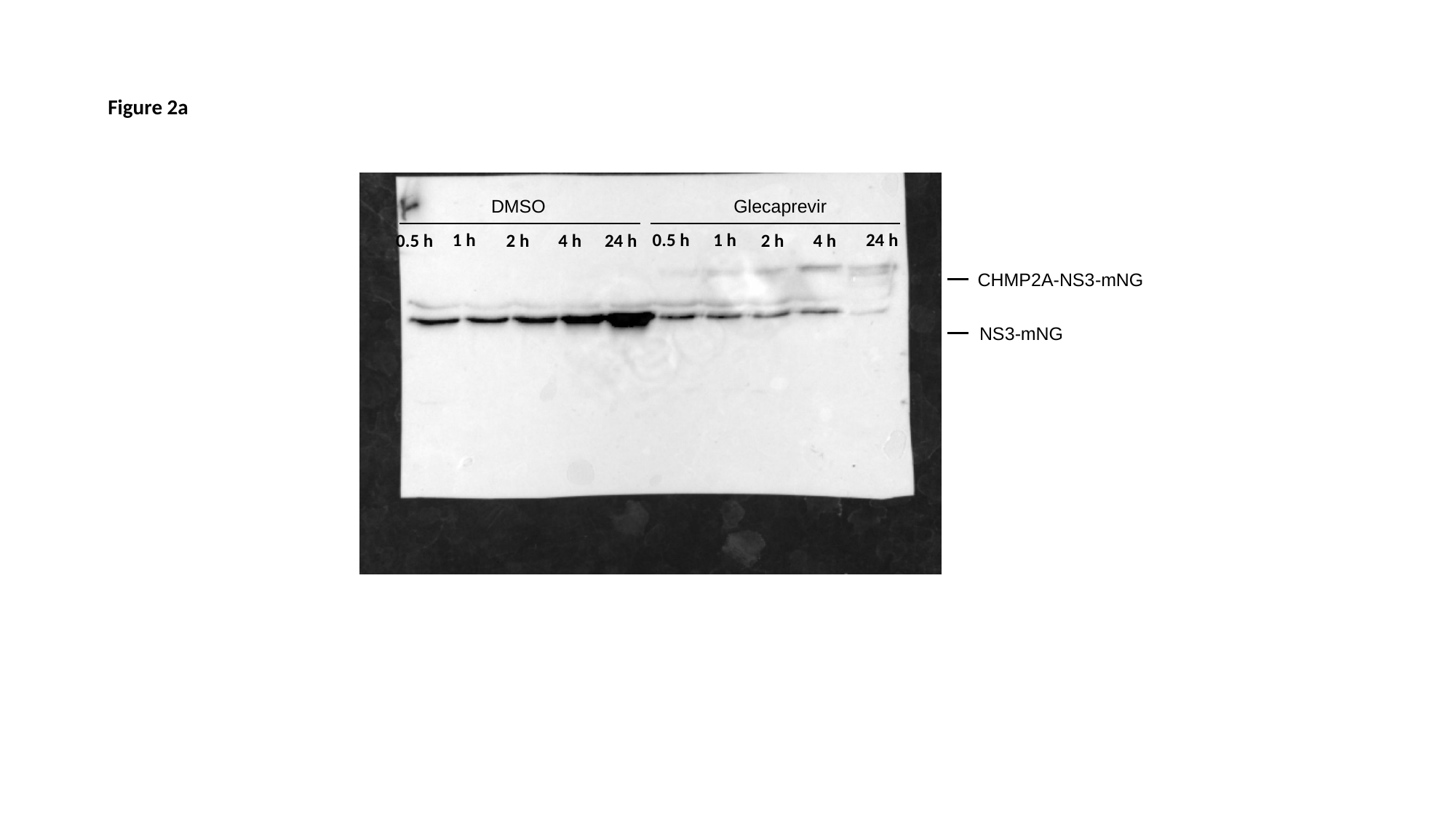

Figure 2a
DMSO
Glecaprevir
1 h
1 h
0.5 h
24 h
0.5 h
24 h
4 h
2 h
4 h
2 h
CHMP2A-NS3-mNG
NS3-mNG

## Slide 2
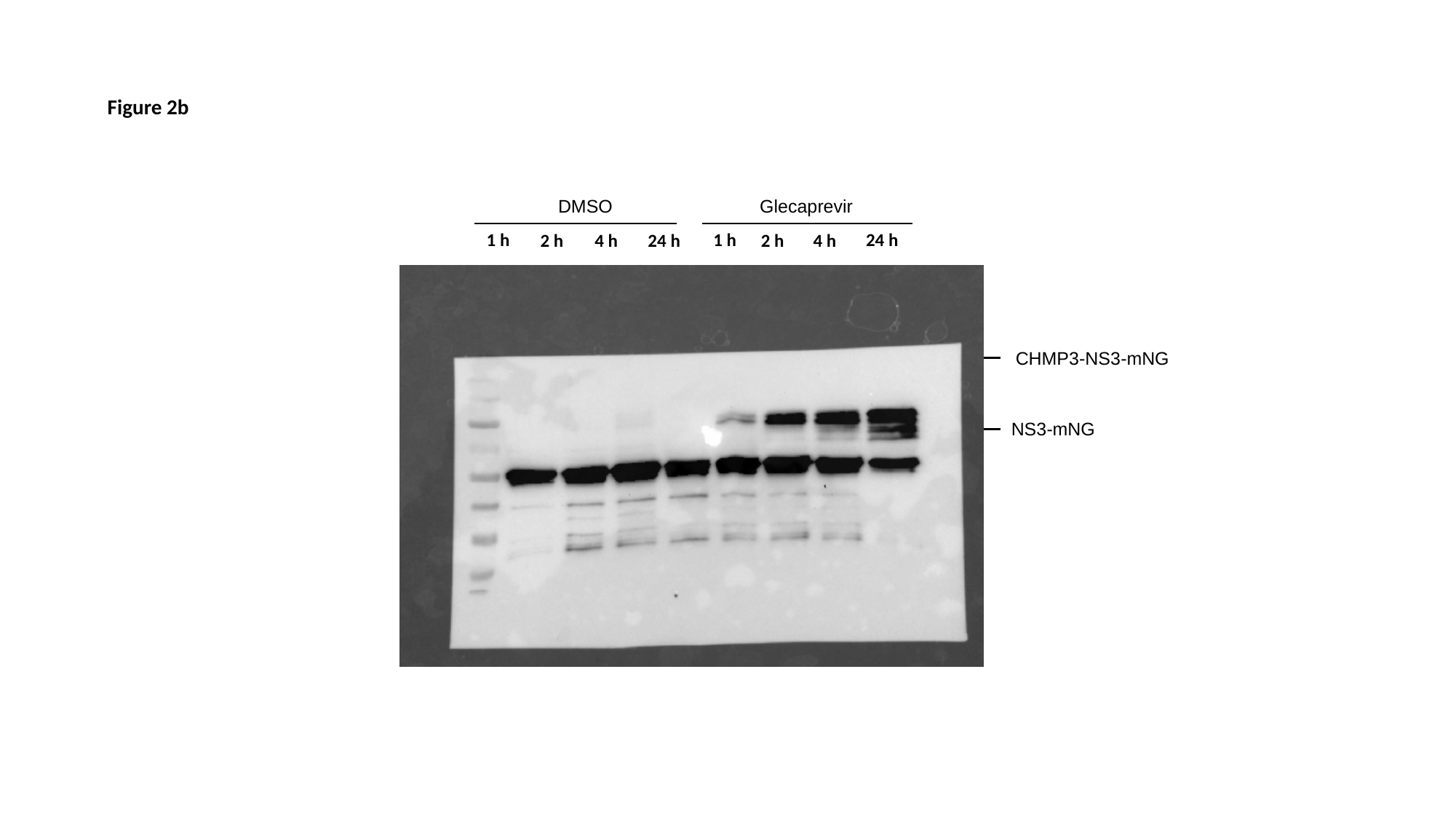

Figure 2b
DMSO
Glecaprevir
1 h
1 h
24 h
24 h
4 h
2 h
4 h
2 h
CHMP3-NS3-mNG
NS3-mNG

## Slide 3
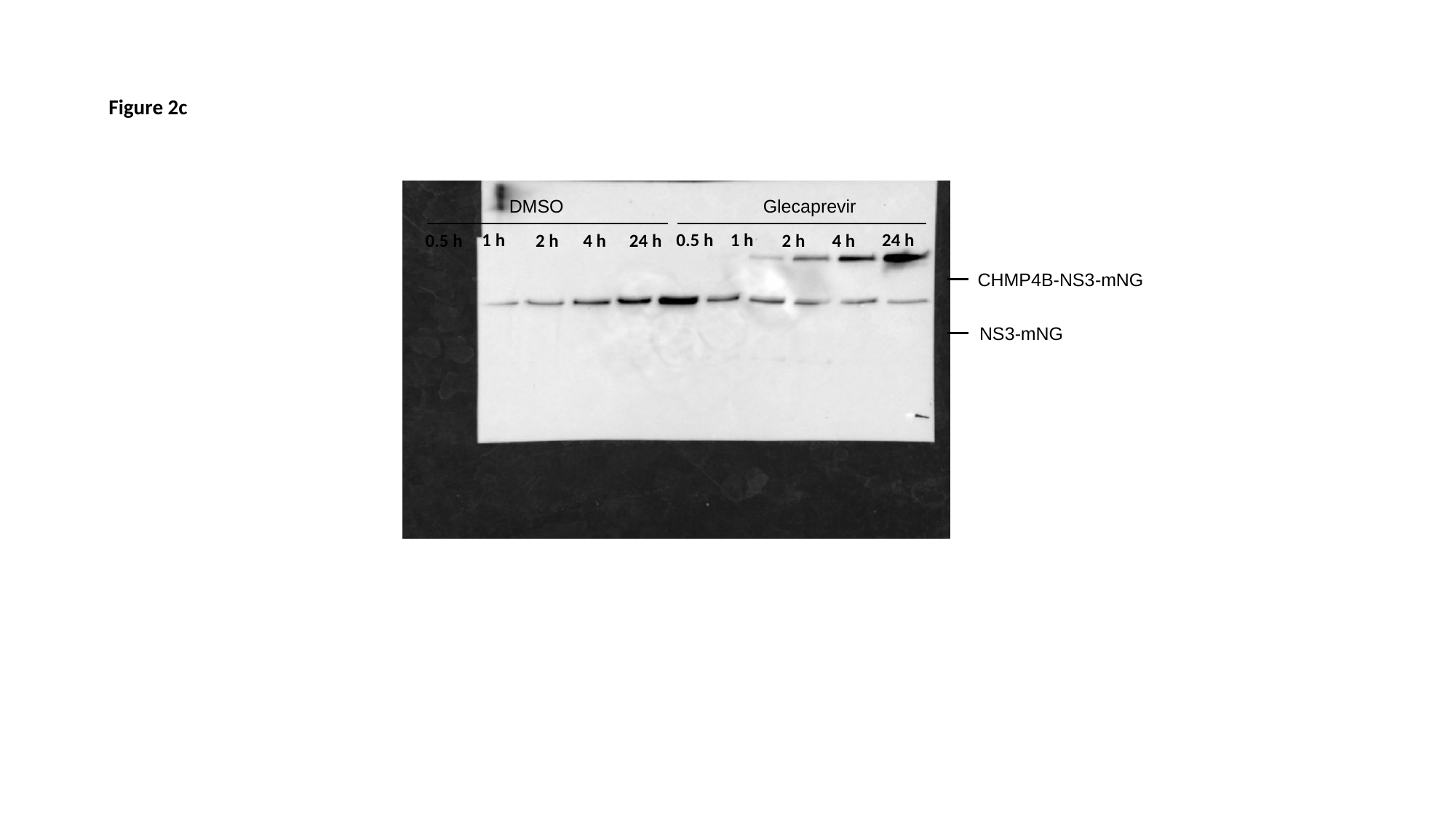

Figure 2c
DMSO
Glecaprevir
1 h
1 h
0.5 h
24 h
0.5 h
24 h
4 h
2 h
4 h
2 h
CHMP4B-NS3-mNG
NS3-mNG

## Slide 4
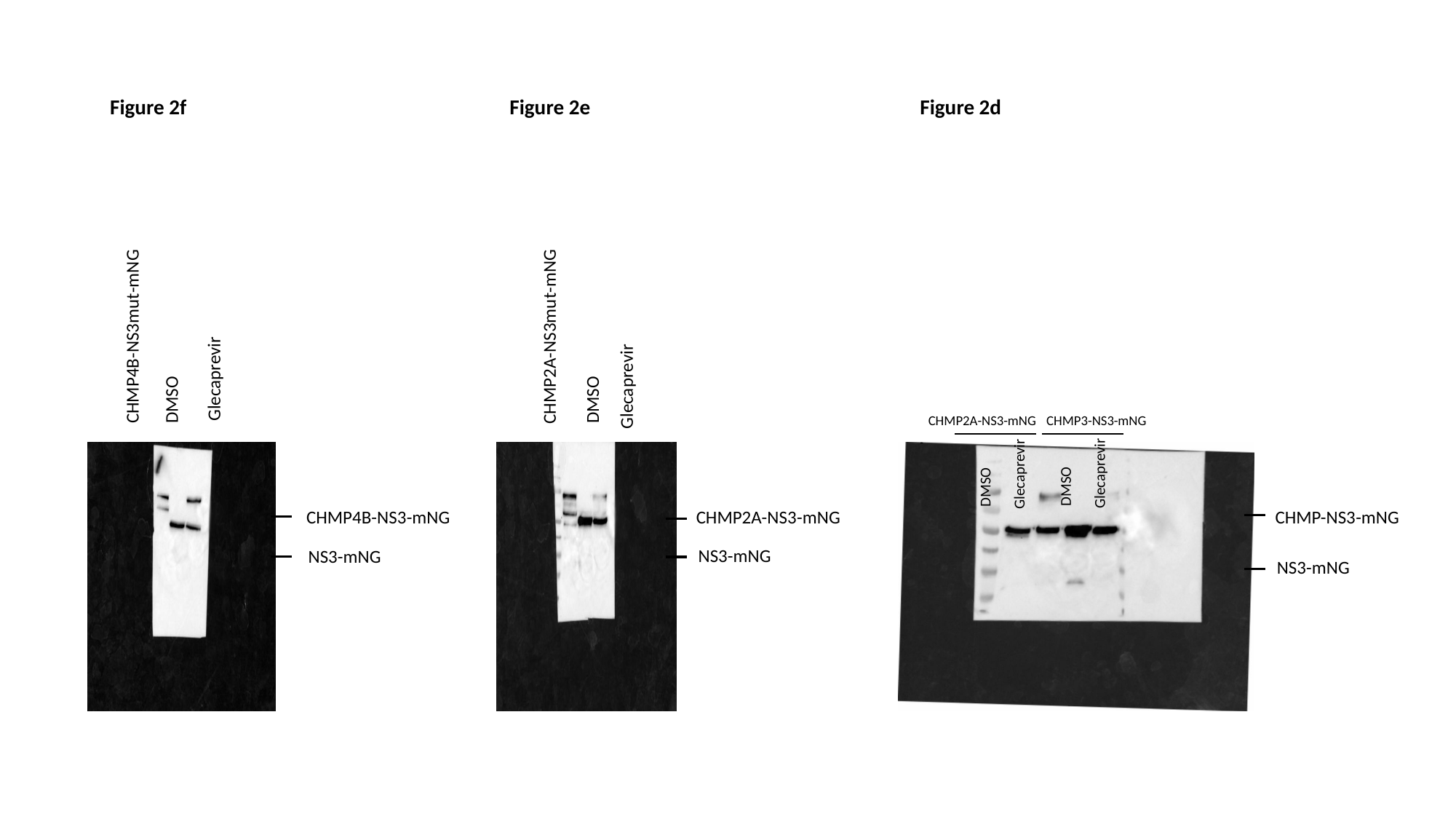

Figure 2f
Figure 2e
Figure 2d
CHMP4B-NS3mut-mNG
CHMP2A-NS3mut-mNG
Glecaprevir
Glecaprevir
DMSO
DMSO
CHMP2A-NS3-mNG
CHMP3-NS3-mNG
Glecaprevir
Glecaprevir
DMSO
DMSO
CHMP4B-NS3-mNG
CHMP-NS3-mNG
CHMP2A-NS3-mNG
NS3-mNG
NS3-mNG
NS3-mNG

## Slide 5
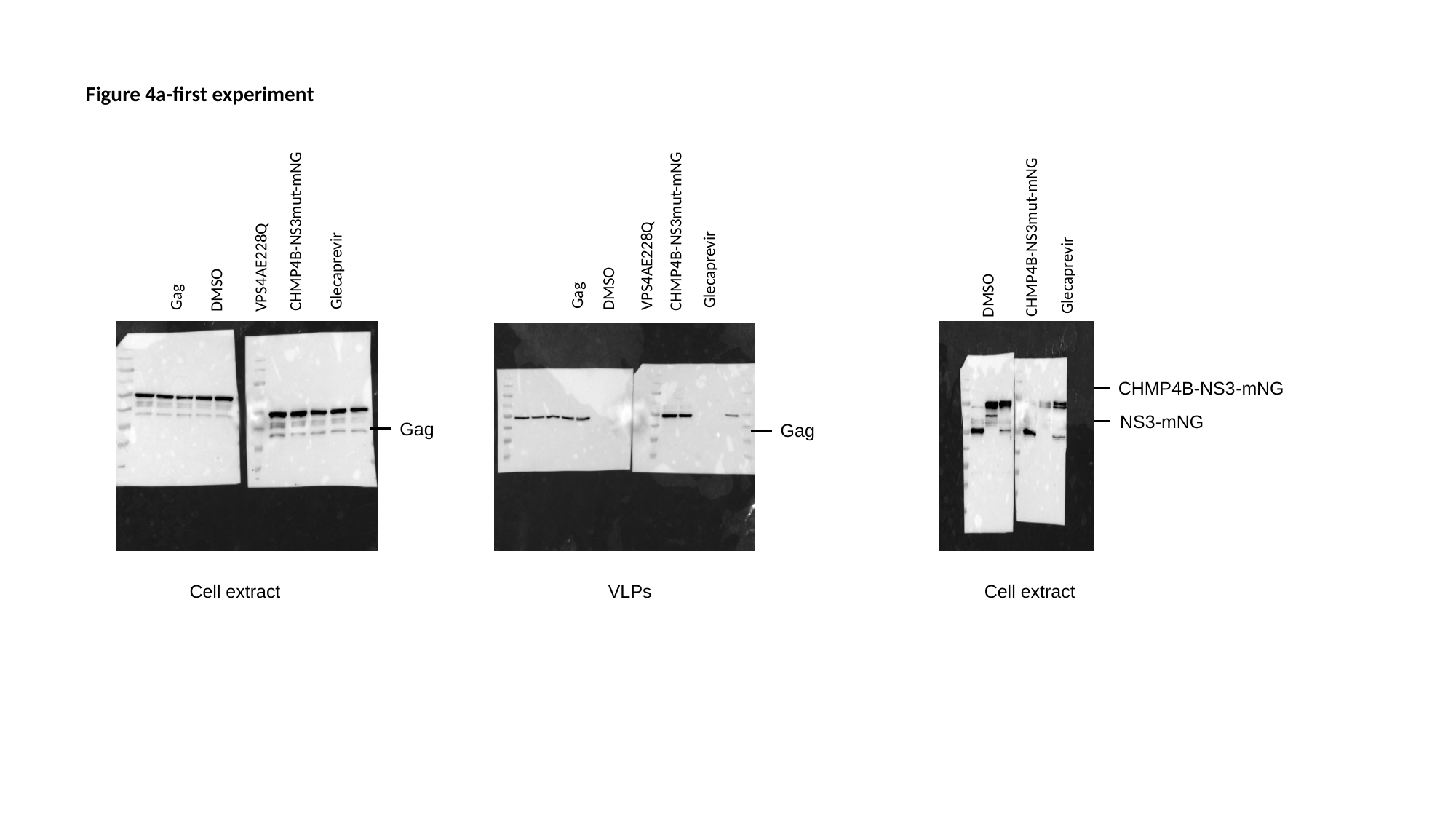

Figure 4a-first experiment
CHMP4B-NS3mut-mNG
CHMP4B-NS3mut-mNG
CHMP4B-NS3mut-mNG
VPS4AE228Q
VPS4AE228Q
Glecaprevir
Glecaprevir
Glecaprevir
DMSO
DMSO
DMSO
Gag
Gag
CHMP4B-NS3-mNG
NS3-mNG
Gag
Gag
Cell extract
Cell extract
VLPs

## Slide 6
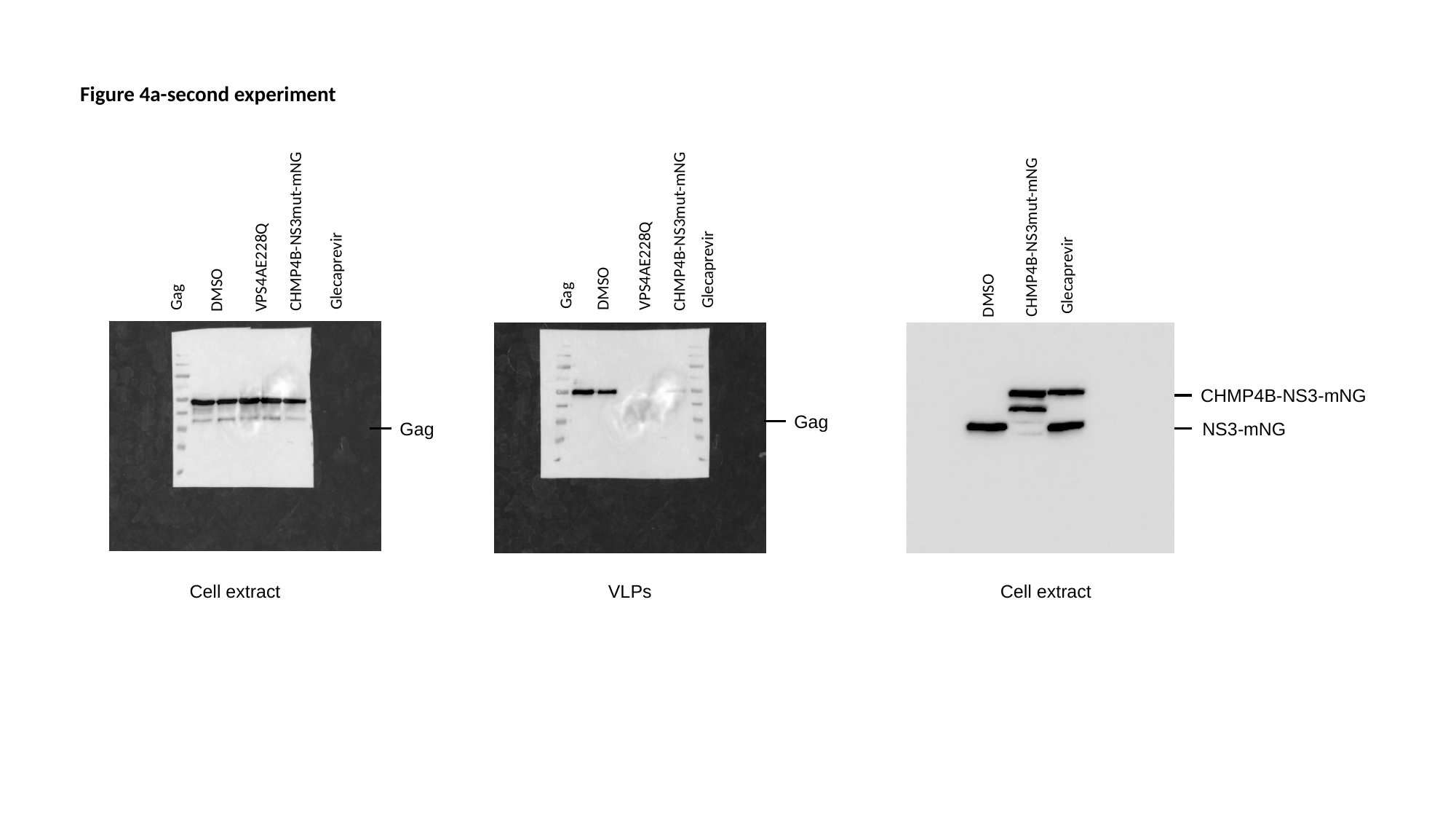

Figure 4a-second experiment
CHMP4B-NS3mut-mNG
CHMP4B-NS3mut-mNG
CHMP4B-NS3mut-mNG
VPS4AE228Q
VPS4AE228Q
Glecaprevir
Glecaprevir
Glecaprevir
DMSO
DMSO
DMSO
Gag
Gag
CHMP4B-NS3-mNG
Gag
Gag
NS3-mNG
Cell extract
VLPs
Cell extract

## Slide 7
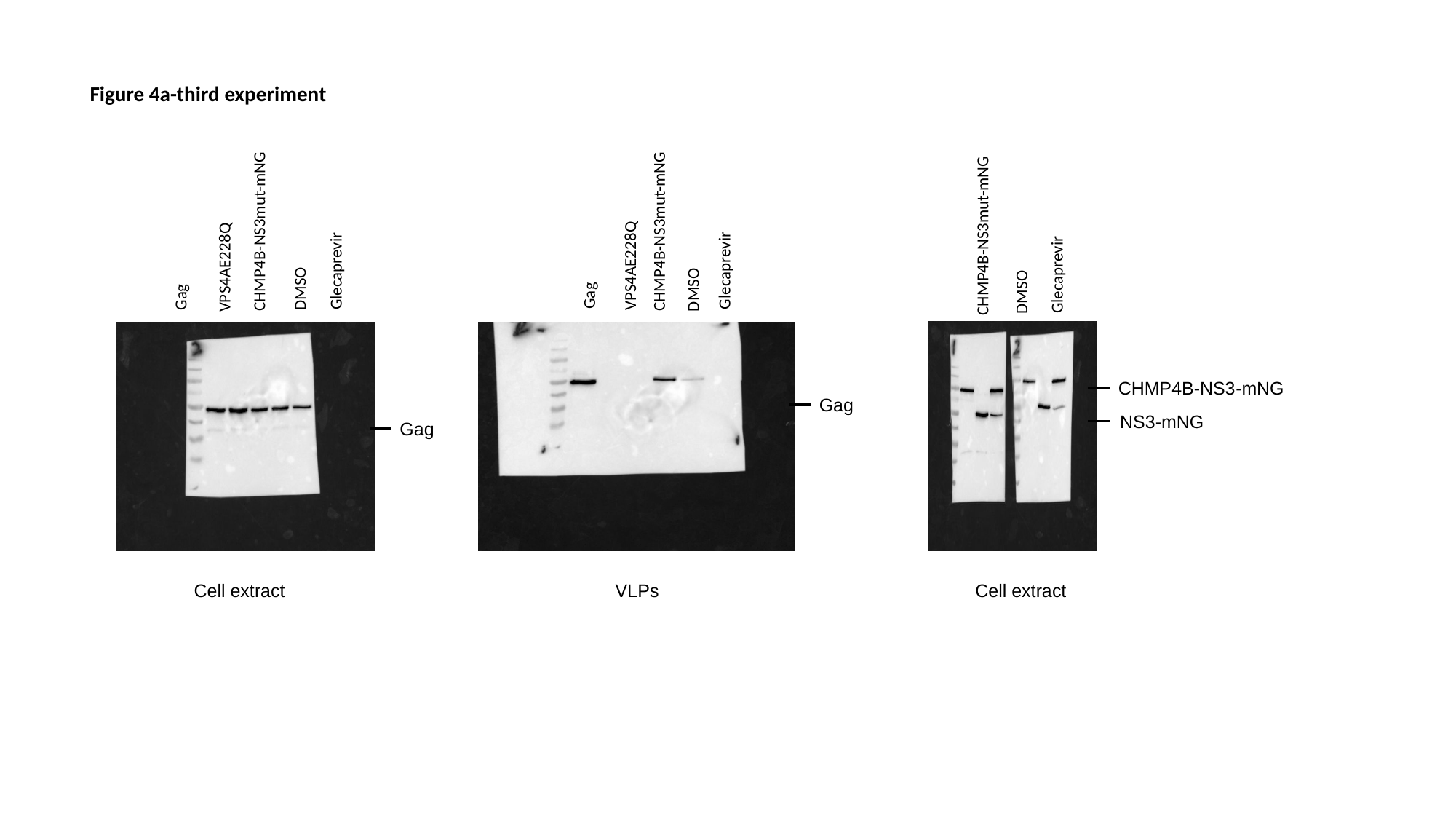

Figure 4a-third experiment
CHMP4B-NS3mut-mNG
CHMP4B-NS3mut-mNG
CHMP4B-NS3mut-mNG
VPS4AE228Q
VPS4AE228Q
Glecaprevir
Glecaprevir
Glecaprevir
DMSO
DMSO
DMSO
Gag
Gag
CHMP4B-NS3-mNG
Gag
NS3-mNG
Gag
Cell extract
Cell extract
VLPs

## Slide 8
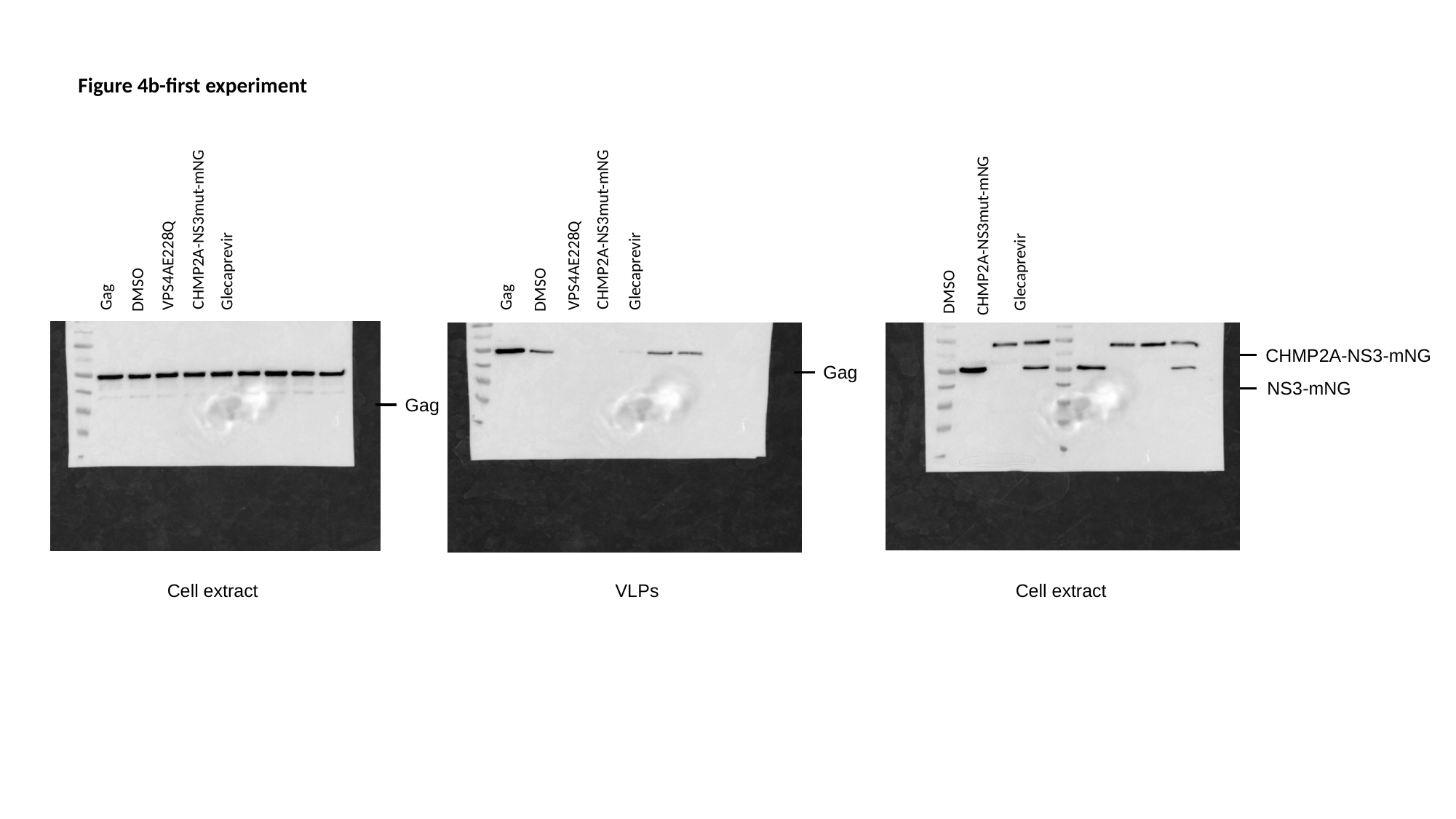

Figure 4b-first experiment
CHMP2A-NS3mut-mNG
CHMP2A-NS3mut-mNG
CHMP2A-NS3mut-mNG
VPS4AE228Q
VPS4AE228Q
Glecaprevir
Glecaprevir
Glecaprevir
DMSO
DMSO
DMSO
Gag
Gag
CHMP2A-NS3-mNG
Gag
NS3-mNG
Gag
Cell extract
Cell extract
VLPs

## Slide 9
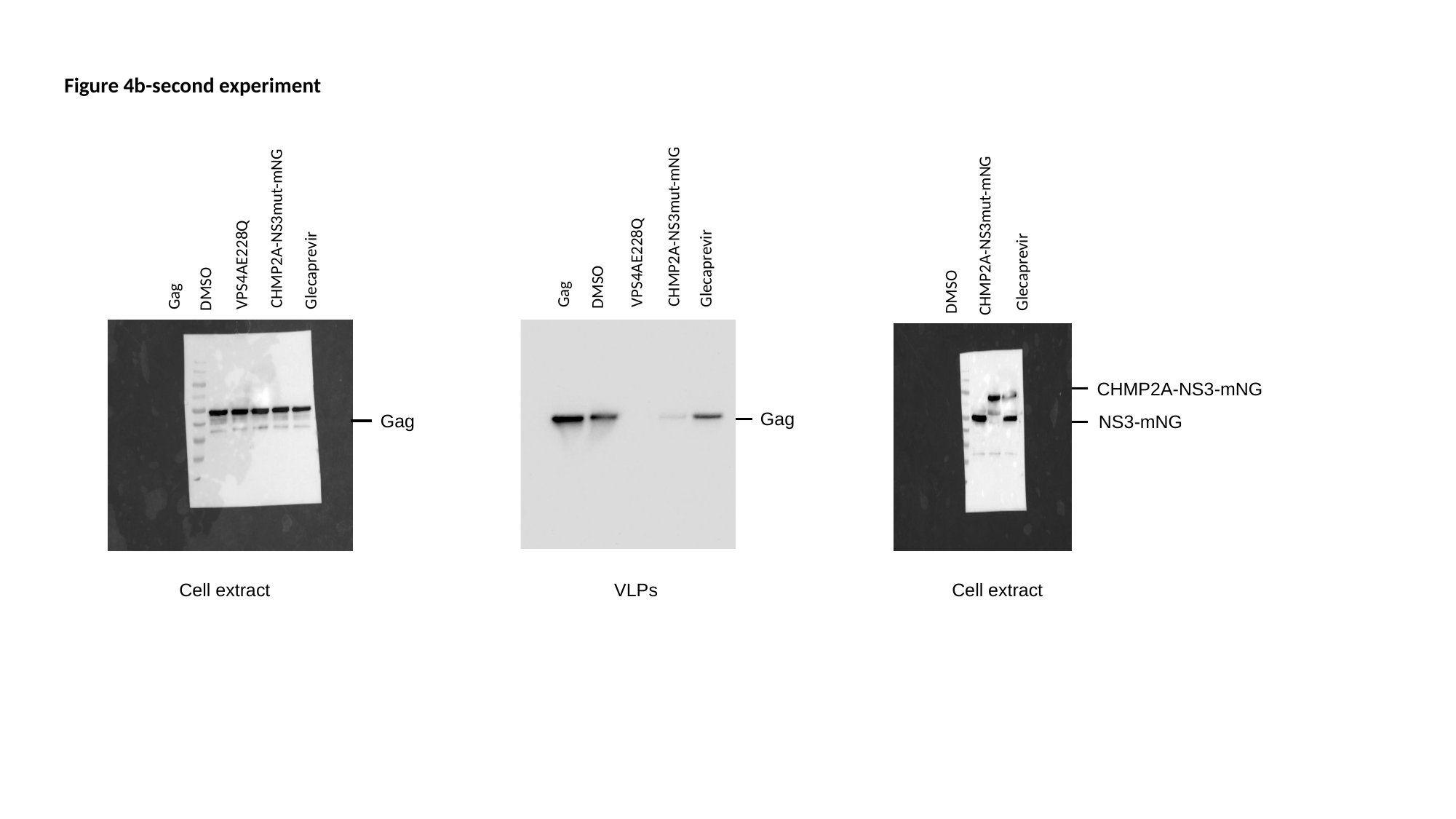

Figure 4b-second experiment
CHMP2A-NS3mut-mNG
CHMP2A-NS3mut-mNG
CHMP2A-NS3mut-mNG
VPS4AE228Q
VPS4AE228Q
Glecaprevir
Glecaprevir
Glecaprevir
DMSO
DMSO
DMSO
Gag
Gag
CHMP2A-NS3-mNG
Gag
Gag
NS3-mNG
Cell extract
VLPs
Cell extract

## Slide 10
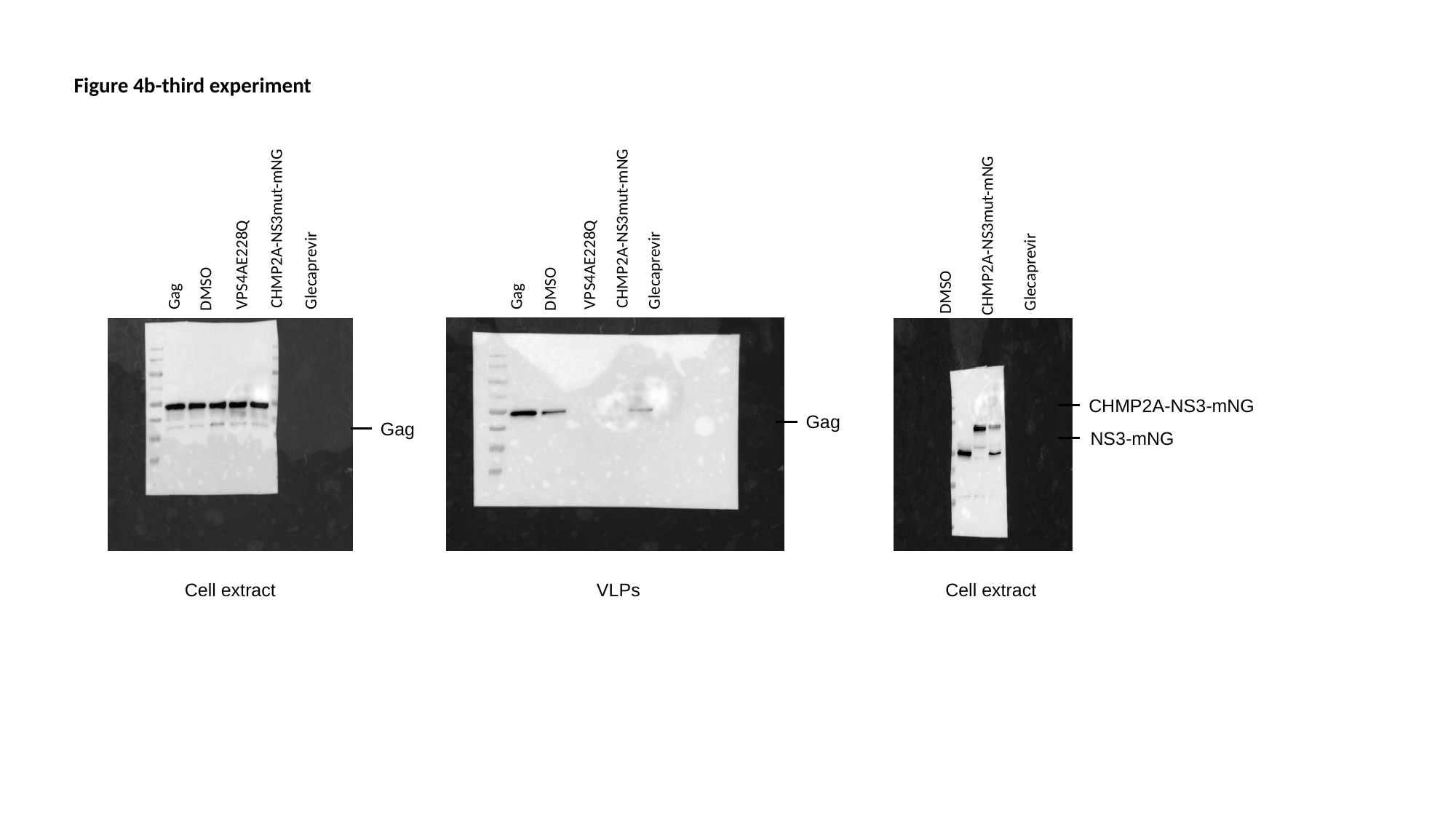

Figure 4b-third experiment
CHMP2A-NS3mut-mNG
CHMP2A-NS3mut-mNG
CHMP2A-NS3mut-mNG
VPS4AE228Q
VPS4AE228Q
Glecaprevir
Glecaprevir
Glecaprevir
DMSO
DMSO
DMSO
Gag
Gag
CHMP2A-NS3-mNG
Gag
Gag
NS3-mNG
Cell extract
VLPs
Cell extract

## Slide 11
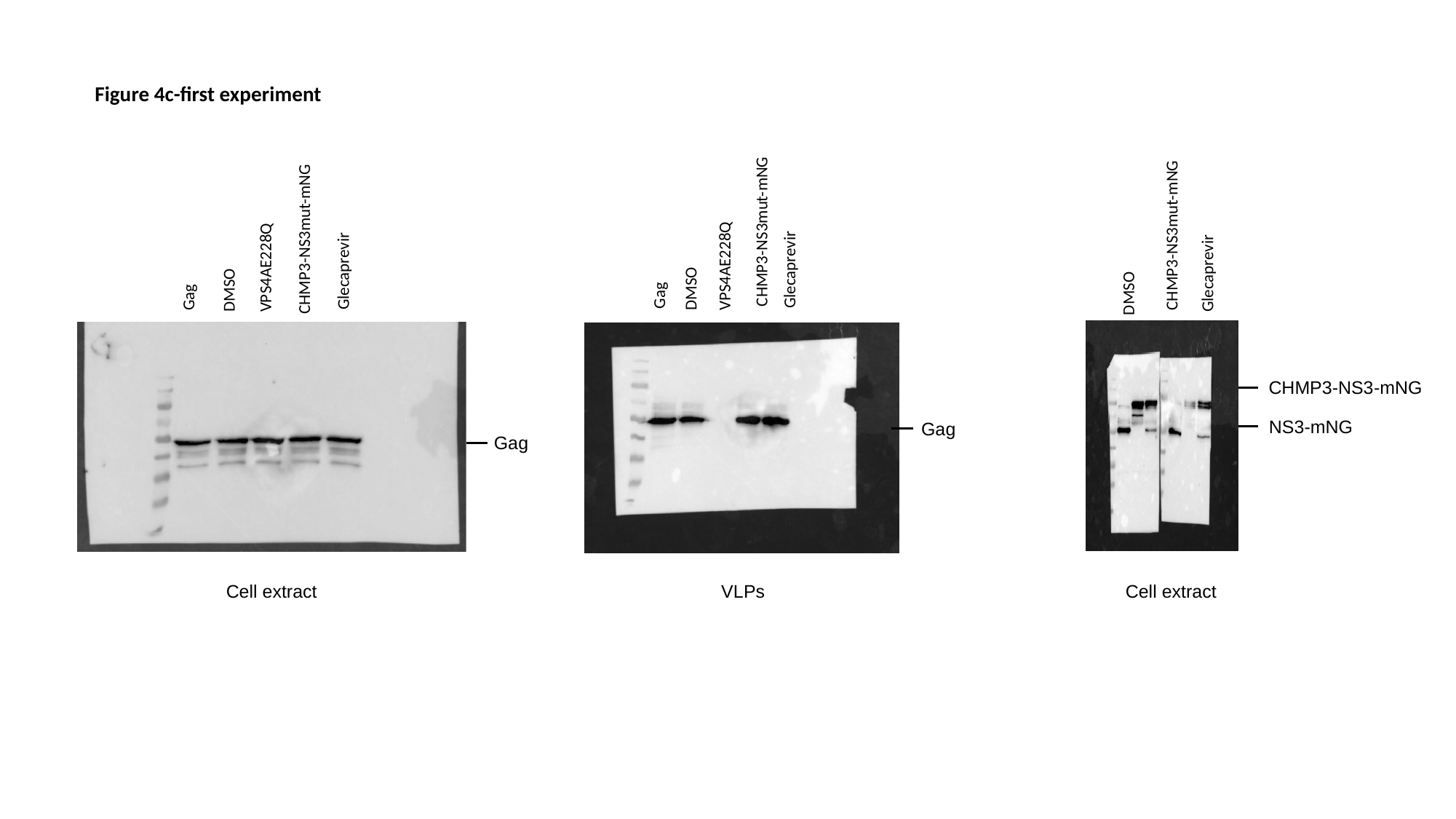

Figure 4c-first experiment
CHMP3-NS3mut-mNG
CHMP3-NS3mut-mNG
CHMP3-NS3mut-mNG
VPS4AE228Q
VPS4AE228Q
Glecaprevir
Glecaprevir
Glecaprevir
DMSO
DMSO
DMSO
Gag
Gag
CHMP3-NS3-mNG
NS3-mNG
Gag
Gag
Cell extract
VLPs
Cell extract

## Slide 12
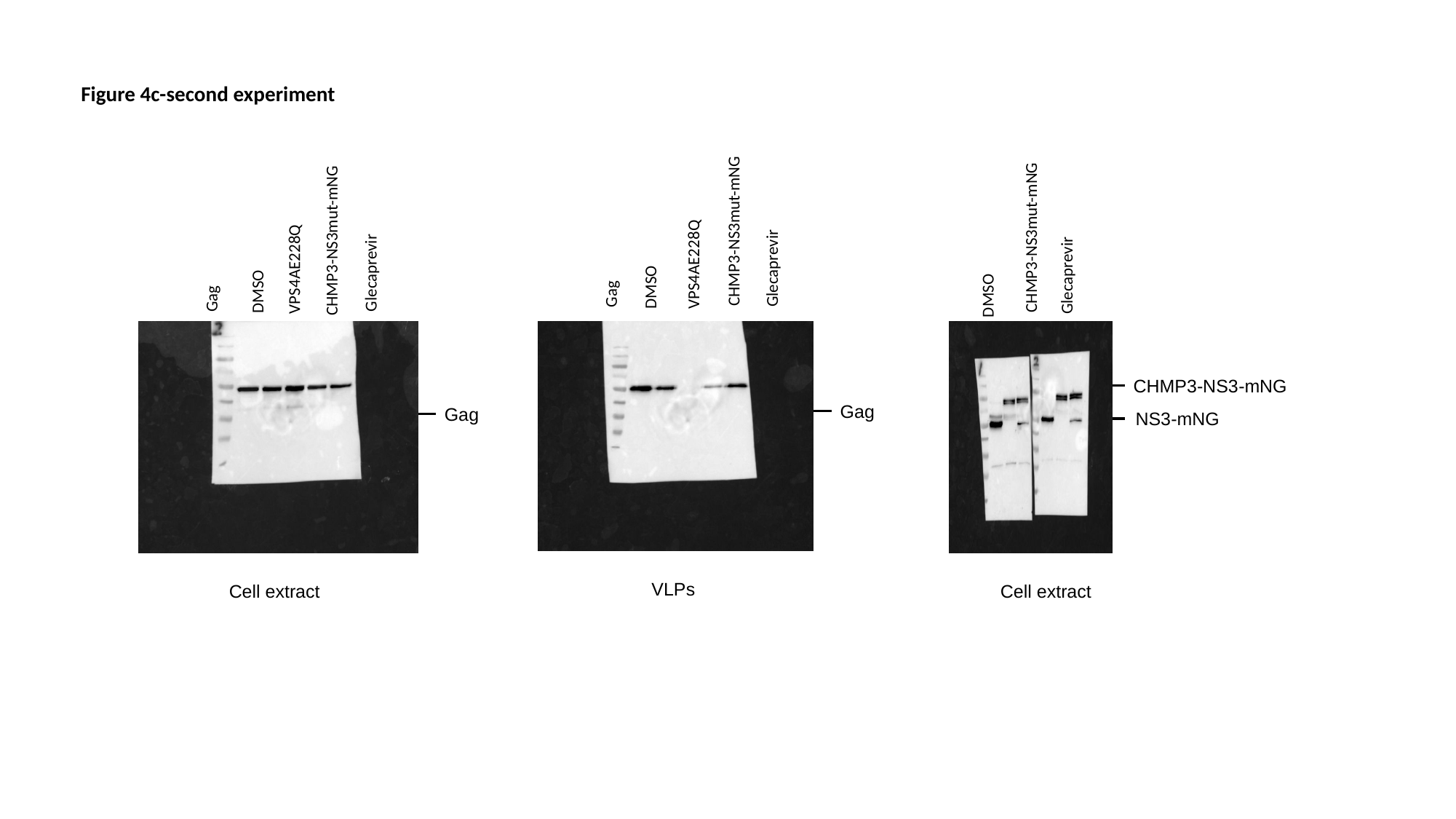

Figure 4c-second experiment
CHMP3-NS3mut-mNG
CHMP3-NS3mut-mNG
CHMP3-NS3mut-mNG
VPS4AE228Q
Glecaprevir
VPS4AE228Q
Glecaprevir
Glecaprevir
DMSO
DMSO
Gag
DMSO
Gag
CHMP3-NS3-mNG
Gag
Gag
NS3-mNG
VLPs
Cell extract
Cell extract

## Slide 13
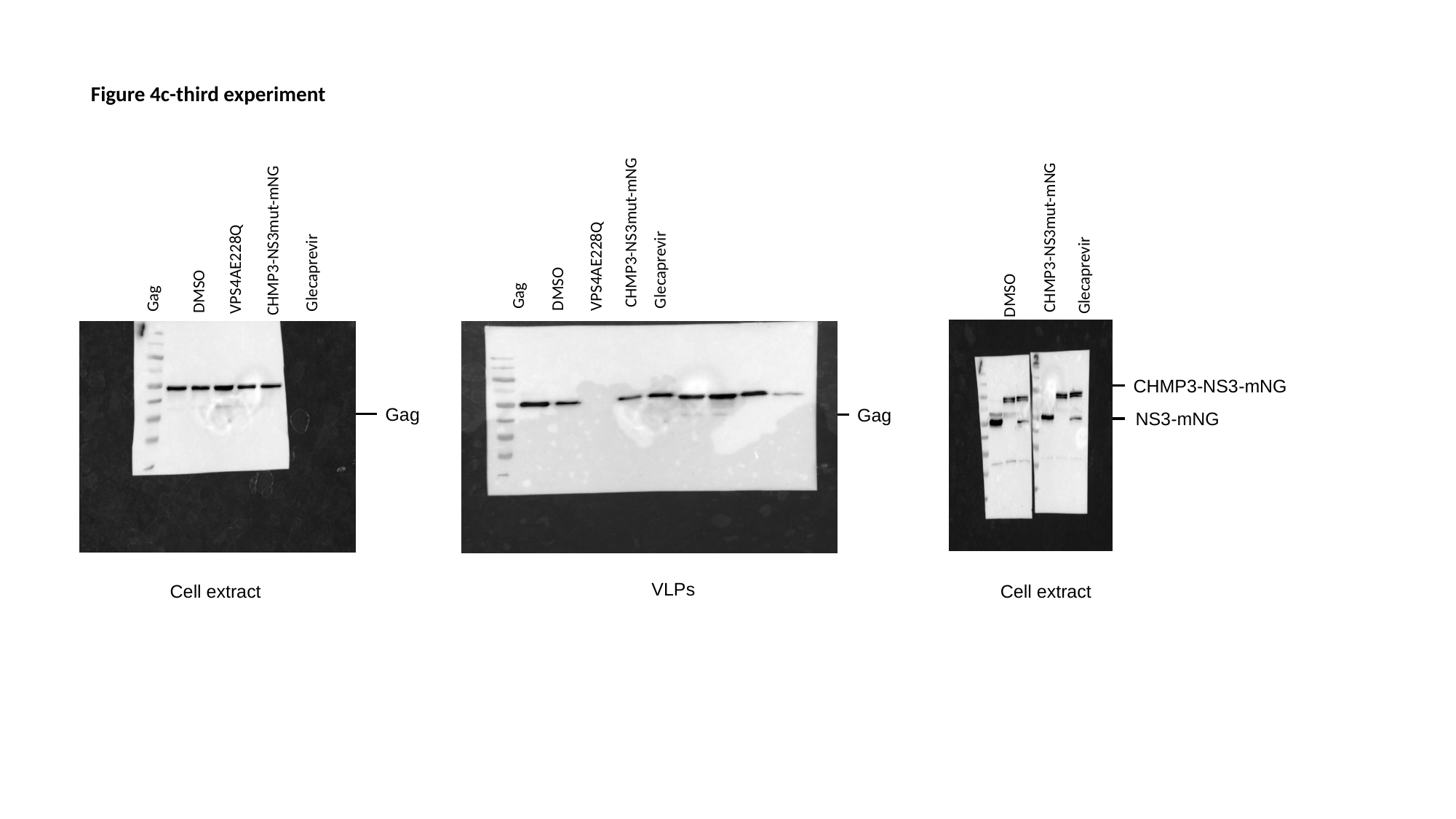

Figure 4c-third experiment
CHMP3-NS3mut-mNG
CHMP3-NS3mut-mNG
CHMP3-NS3mut-mNG
VPS4AE228Q
VPS4AE228Q
Glecaprevir
Glecaprevir
Glecaprevir
DMSO
DMSO
DMSO
Gag
Gag
CHMP3-NS3-mNG
Gag
Gag
NS3-mNG
VLPs
Cell extract
Cell extract

## Slide 14
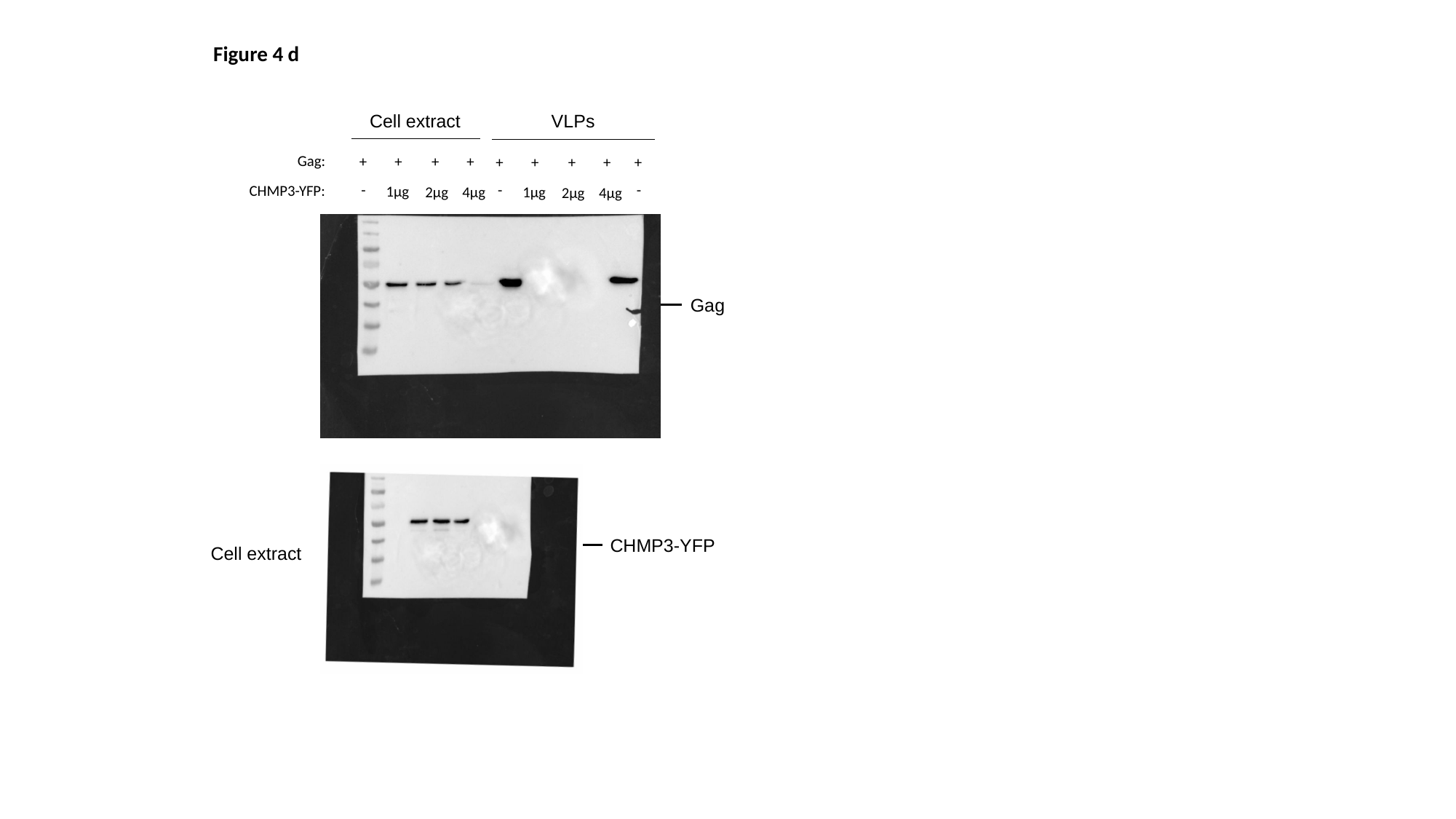

Figure 4 d
Cell extract
VLPs
Gag:
+
+
+
+
+
+
+
+
+
-
-
-
CHMP3-YFP:
1µg
1µg
2µg
4µg
2µg
4µg
Gag
CHMP3-YFP
Cell extract

## Slide 15
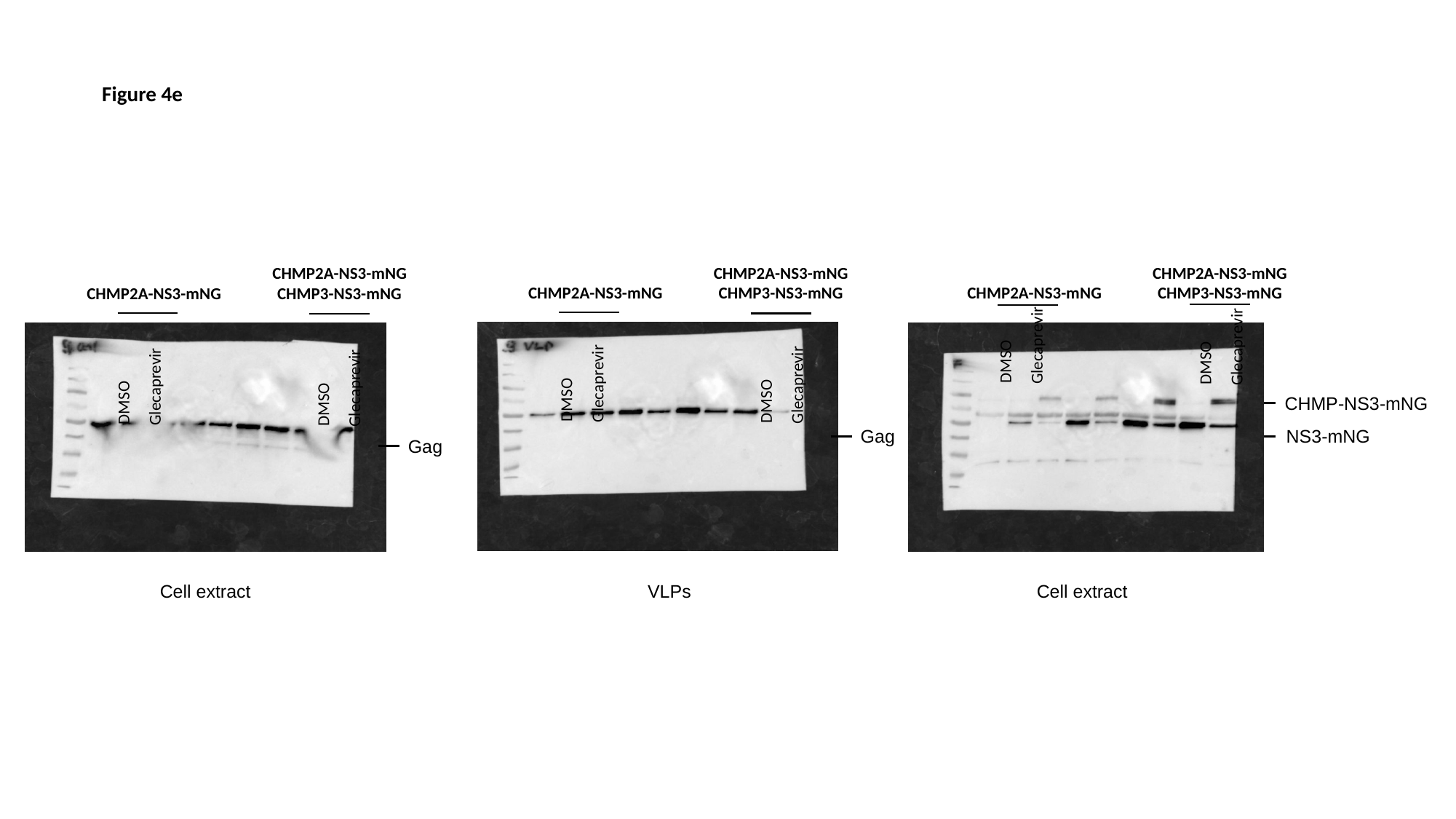

Figure 4e
CHMP2A-NS3-mNG
CHMP3-NS3-mNG
CHMP2A-NS3-mNG
CHMP3-NS3-mNG
CHMP2A-NS3-mNG
CHMP3-NS3-mNG
CHMP2A-NS3-mNG
CHMP2A-NS3-mNG
CHMP2A-NS3-mNG
Glecaprevir
Glecaprevir
DMSO
DMSO
Glecaprevir
Glecaprevir
Glecaprevir
Glecaprevir
DMSO
DMSO
CHMP-NS3-mNG
DMSO
DMSO
Gag
NS3-mNG
Gag
Cell extract
VLPs
Cell extract

## Slide 16
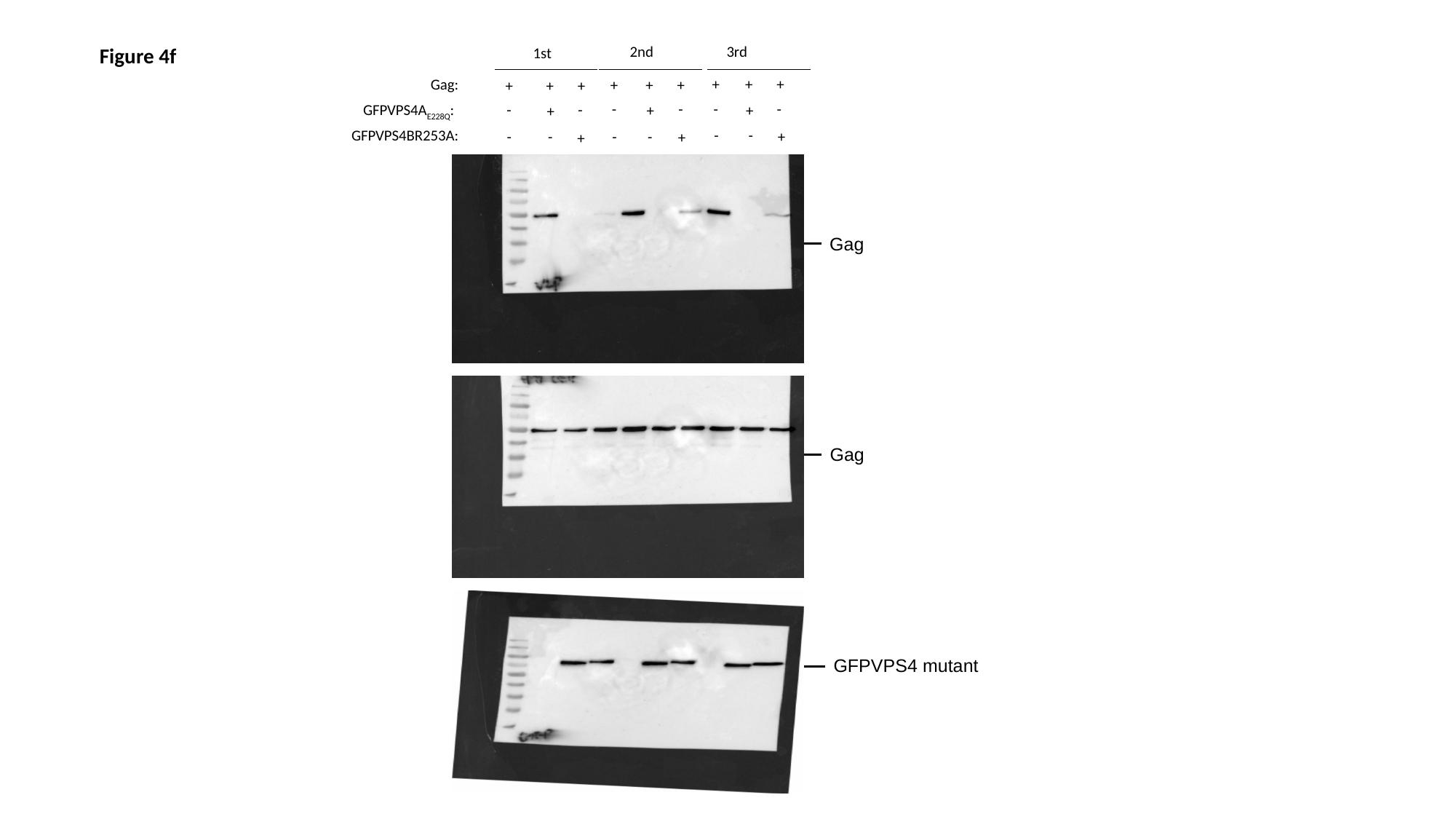

3rd
2nd
Figure 4f
1st
Gag:
+
+
+
+
+
+
+
+
+
-
-
-
-
-
-
GFPVPS4AE228Q:
+
+
+
GFPVPS4BR253A:
-
-
+
-
-
-
-
+
+
Gag
Gag
GFPVPS4 mutant

## Slide 17
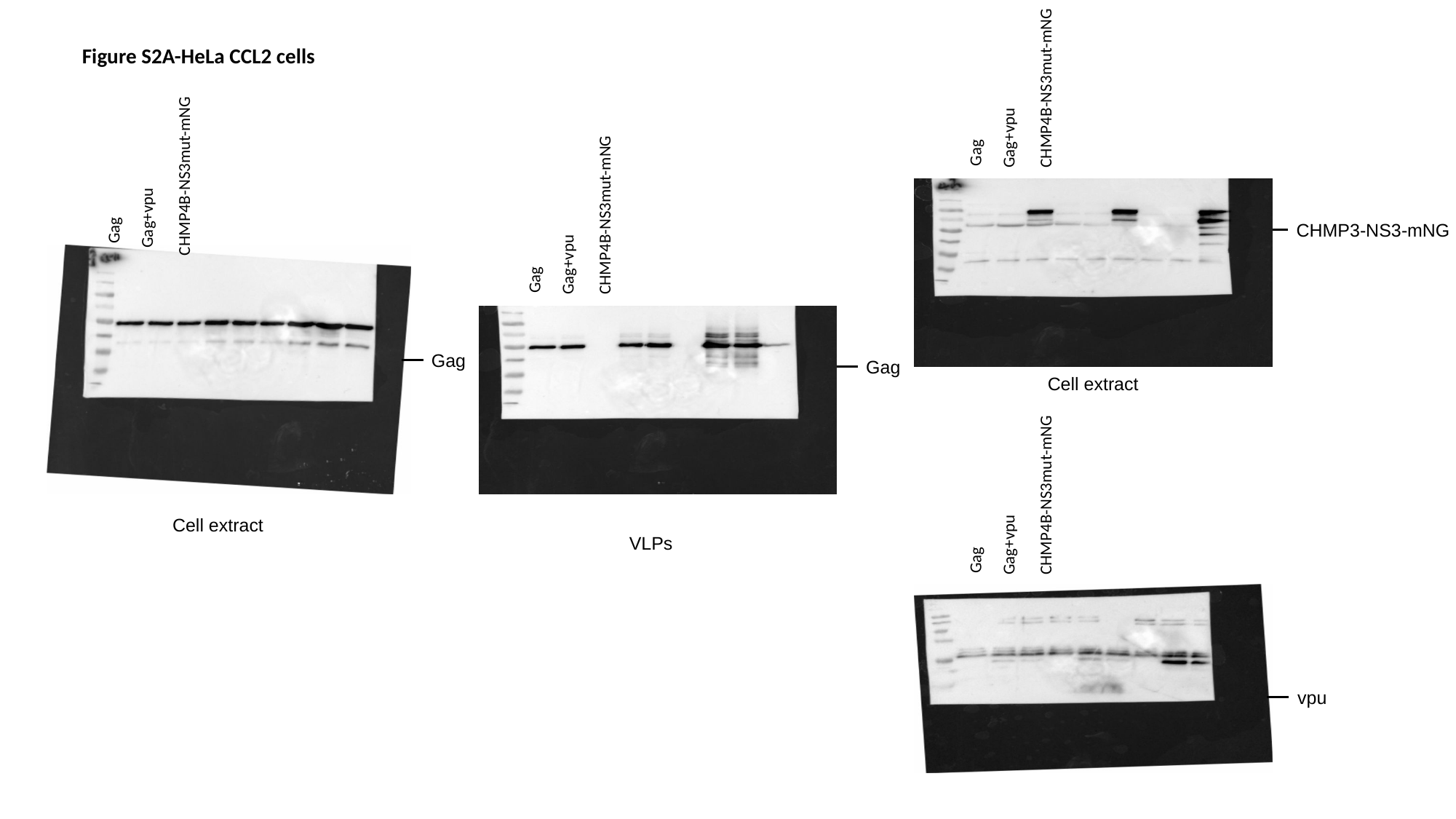

Figure S2A-HeLa CCL2 cells
CHMP4B-NS3mut-mNG
Gag+vpu
Gag
CHMP4B-NS3mut-mNG
CHMP4B-NS3mut-mNG
Gag+vpu
Gag
CHMP3-NS3-mNG
Gag+vpu
Gag
Gag
Gag
Cell extract
CHMP4B-NS3mut-mNG
Cell extract
VLPs
Gag+vpu
Gag
vpu

## Slide 18
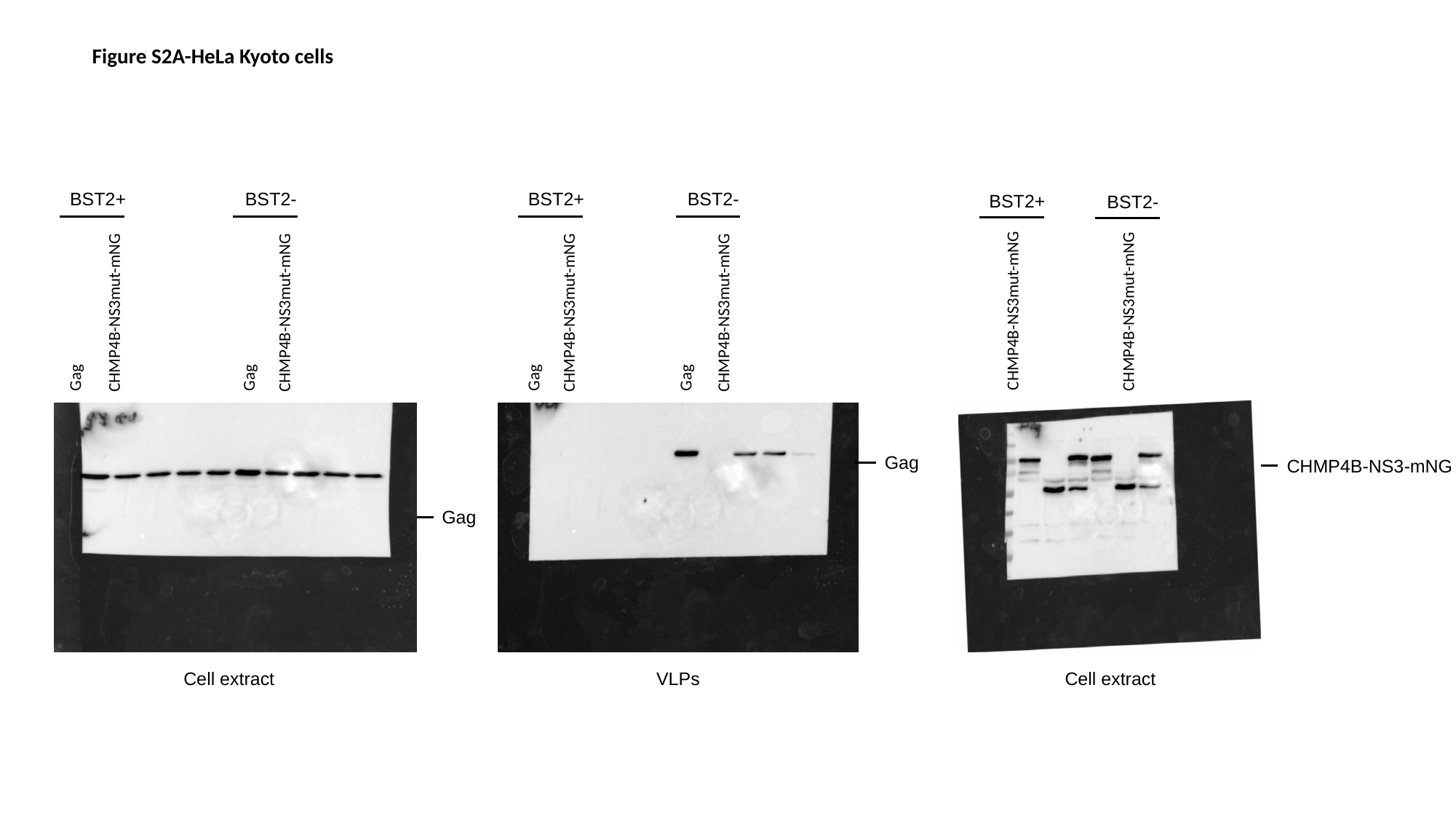

Figure S2A-HeLa Kyoto cells
BST2-
BST2-
BST2+
BST2+
BST2+
BST2-
CHMP4B-NS3mut-mNG
CHMP4B-NS3mut-mNG
CHMP4B-NS3mut-mNG
CHMP4B-NS3mut-mNG
CHMP4B-NS3mut-mNG
CHMP4B-NS3mut-mNG
Gag
Gag
Gag
Gag
Gag
CHMP4B-NS3-mNG
Gag
Cell extract
VLPs
Cell extract

## Slide 19
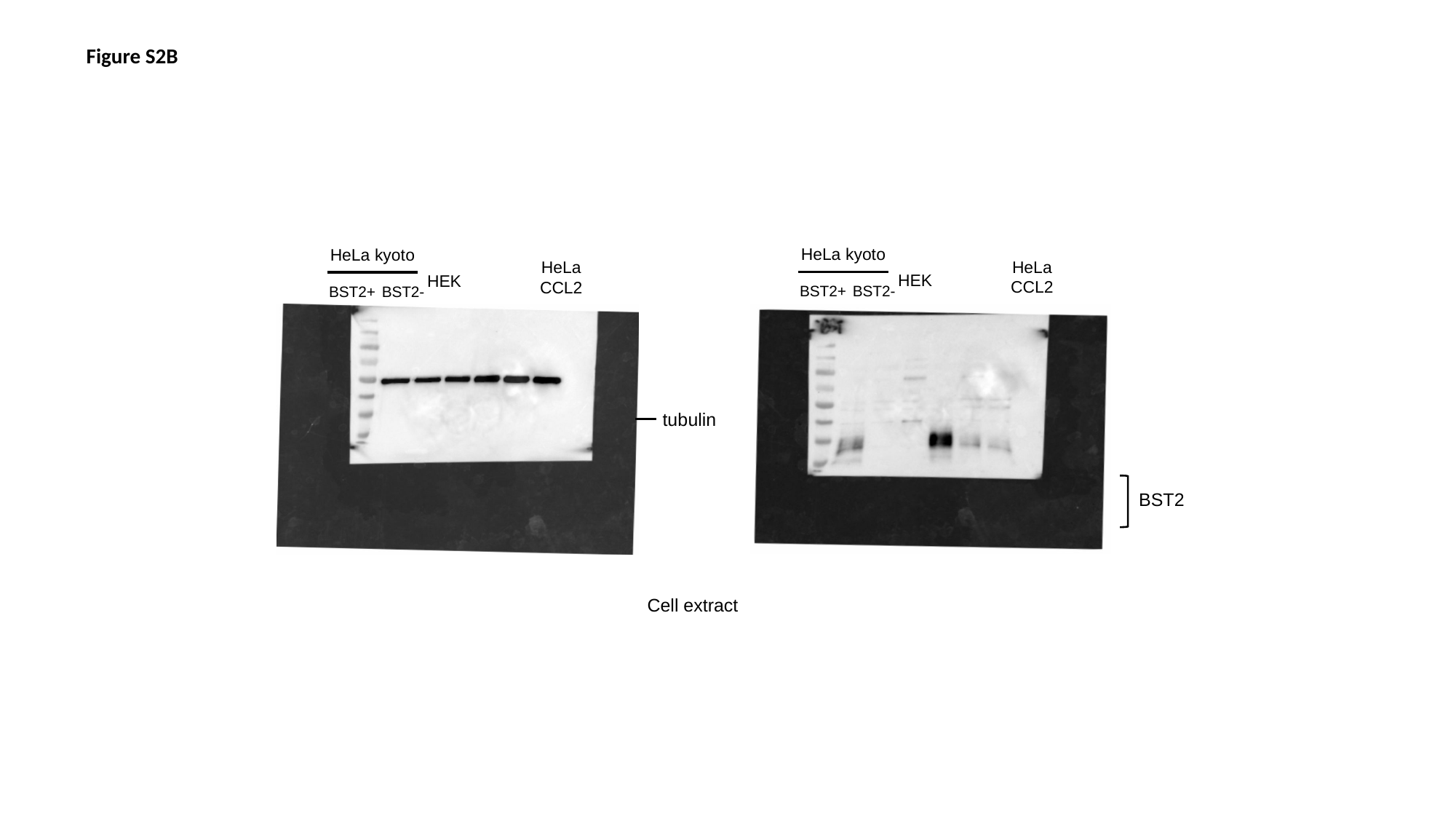

Figure S2B
HeLa kyoto
HeLa kyoto
HeLa
CCL2
HeLa
CCL2
HEK
HEK
BST2-
BST2+
BST2-
BST2+
tubulin
BST2
Cell extract

## Slide 20
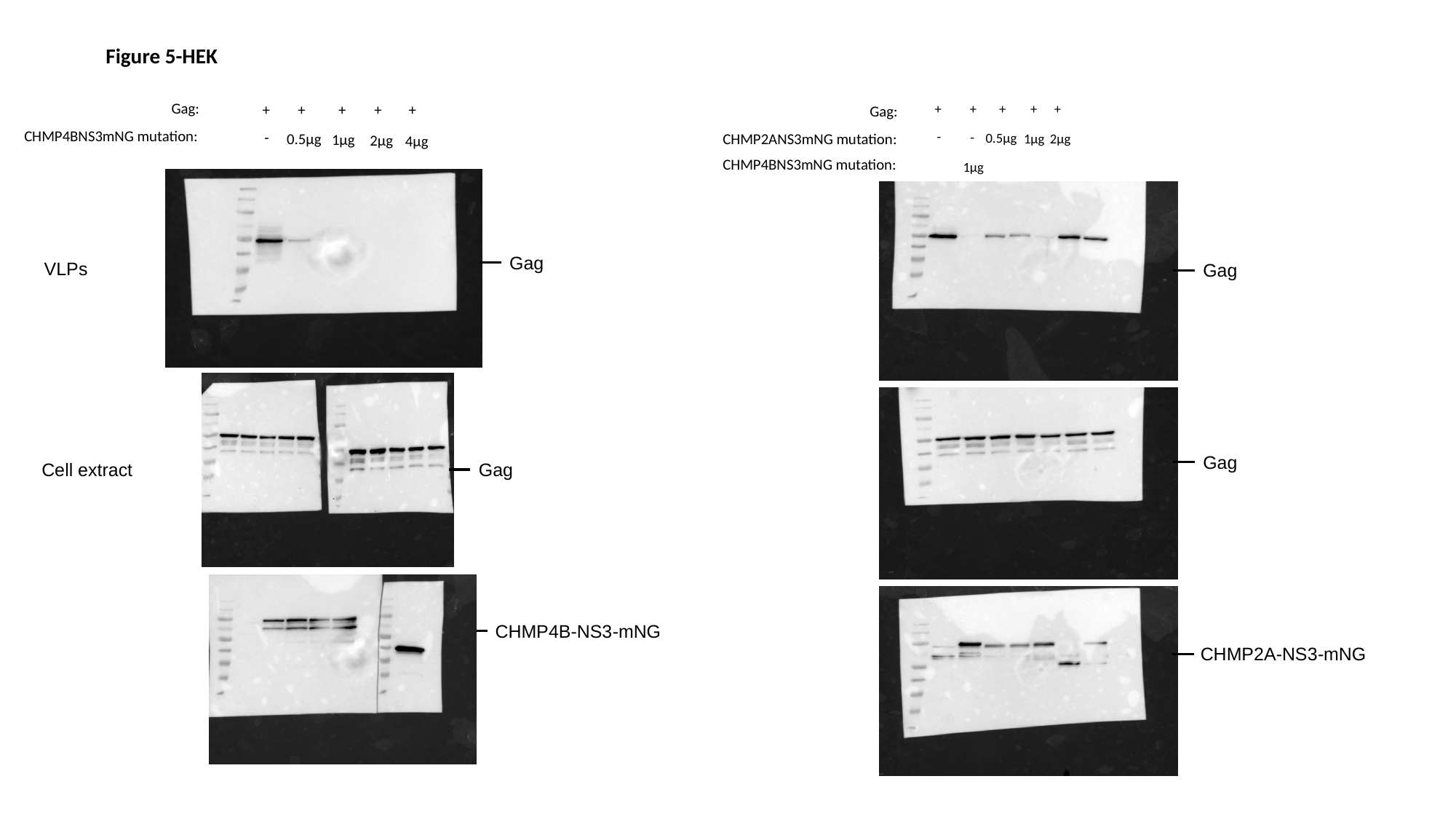

Figure 5-HEK
Gag:
+
+
+
+
+
+
+
+
+
+
Gag:
CHMP4BNS3mNG mutation:
-
-
-
CHMP2ANS3mNG mutation:
0.5µg
0.5µg
1µg
1µg
2µg
2µg
4µg
CHMP4BNS3mNG mutation:
1µg
Gag
VLPs
Gag
Gag
Cell extract
Gag
CHMP4B-NS3-mNG
CHMP2A-NS3-mNG

## Slide 21
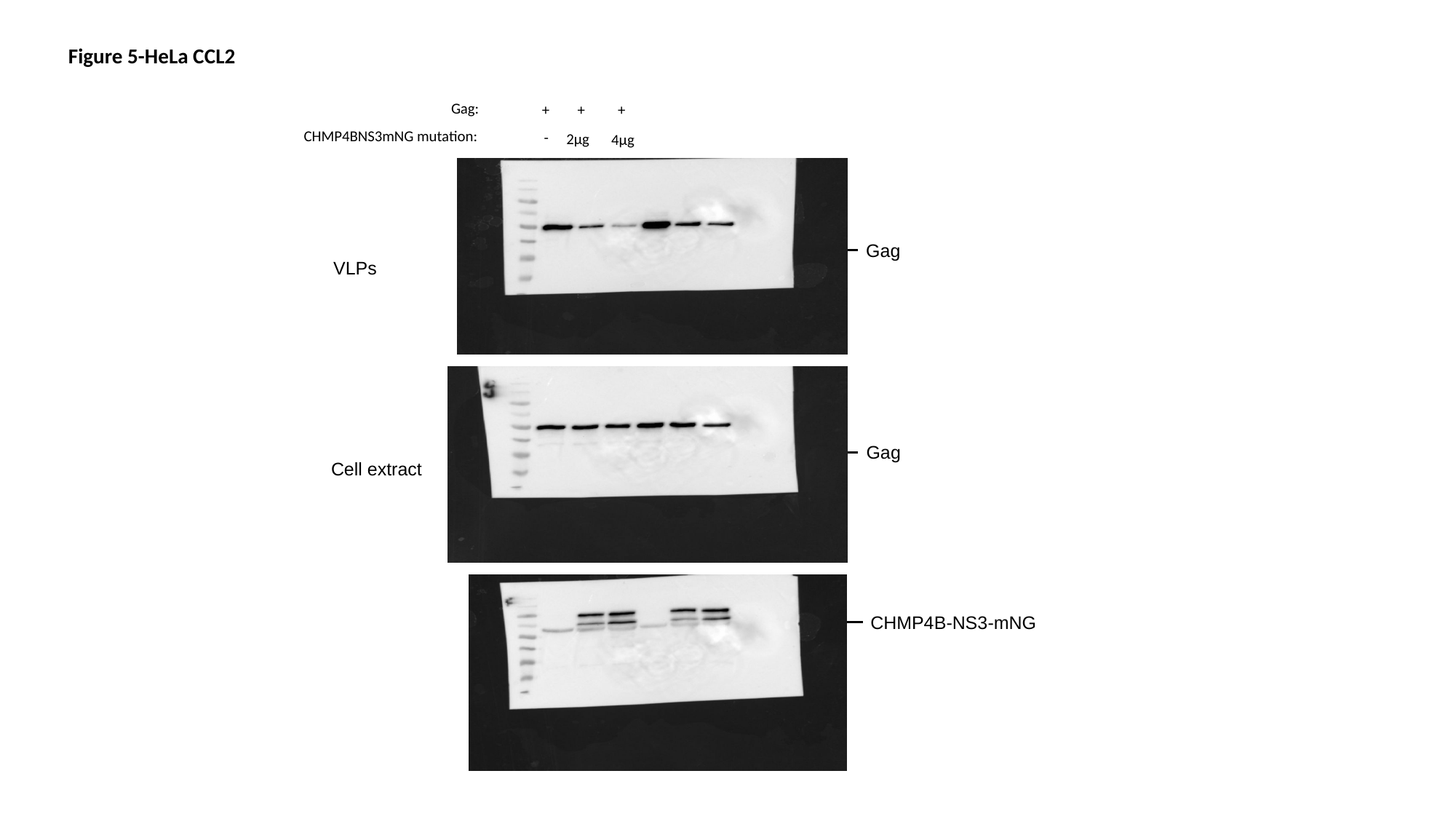

Figure 5-HeLa CCL2
Gag:
+
+
+
CHMP4BNS3mNG mutation:
-
2µg
4µg
Gag
VLPs
Gag
Cell extract
CHMP4B-NS3-mNG

## Slide 22
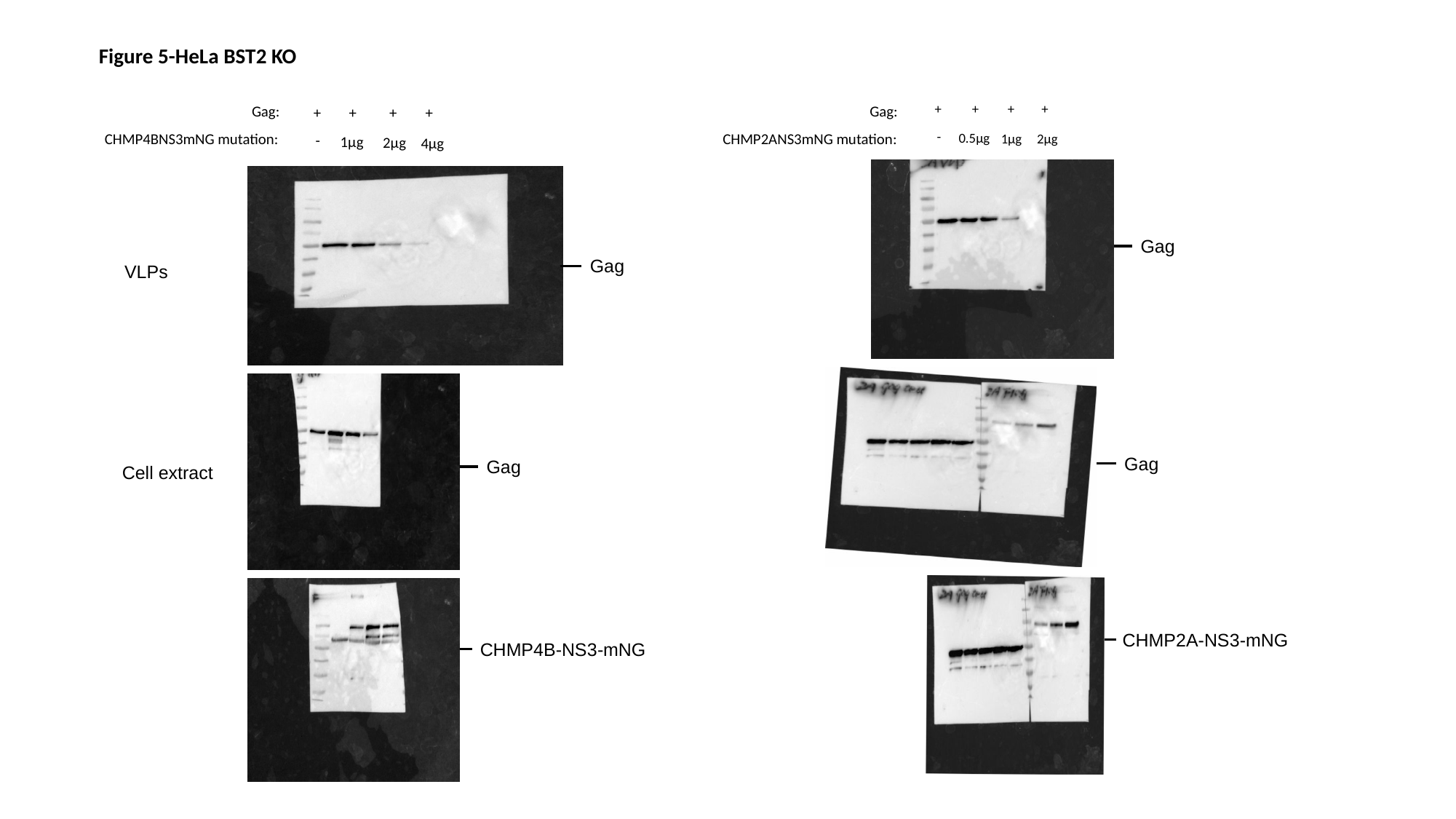

Figure 5-HeLa BST2 KO
+
+
+
+
Gag:
Gag:
+
+
+
+
-
CHMP4BNS3mNG mutation:
CHMP2ANS3mNG mutation:
0.5µg
1µg
2µg
-
1µg
2µg
4µg
Gag
Gag
VLPs
Gag
Gag
Cell extract
CHMP2A-NS3-mNG
CHMP4B-NS3-mNG
