# Supplementary material for: An Inducible ESCRT-III Inhibition Tool to Control HIV-1 Budding
Source: Viruses. 2023 Nov 22;15(12):2289. doi: 10.3390/v15122289 (PMC10748027; doi:10.3390/v15122289)
Supplement: Supplementary file 1 [file viruses-15-02289-s001.zip › Table S1.pdf]

**Table S1: Summary of CHMP fusion protein sequence**	
**Construct name**	**Sequence**
CHMP2A-NS3	CHMP2A(1-222)–××××–DEMEECSQHL–××–NS3/4A(1-197)–××–Flag
CHMP3 -NS3	CHMP3 (1-222)–××××–DEMEECSQHL–××–NS3/4A(1-197)–××–Flag
CHMP4B-NS3	CHMP4B(1-224)–××××–DEMEECSQHL–××–NS3/4A(1-197)–××–Flag
CHMP2A-NS3-blue	CHMP2A(1-222)–××××–DEMEECSQHL–××–NS3/4A(1-197)–××–Flag–××××–mTurquoise(1-238)
CHMP3 -NS3-blue	CHMP3 (1-222)–××××–DEMEECSQHL–××–NS3/4A(1-197)–××–Flag–××××–mTurquoise(1-238)
CHMP4B-NS3-blue	CHMP4B(1-224)–××××–DEMEECSQHL–××–NS3/4A(1-197)–××–Flag–××××–mTurquoise(1-238)
CHMP2A-NS3-green	CHMP2A(1-222)–××××–DEMEECSQHL–××–NS3/4A(1-197)–××–Flag–××–mNeonGreen(1-236)
CHMP3 -NS3-green	CHMP3 (1-222)–××××–DEMEECSQHL–××–NS3/4A(1-197)–××–Flag–××–mNeonGreen(1-236)
CHMP4B-NS3-green	CHMP4B(1-224)–××××–DEMEECSQHL–××–NS3/4A(1-197)–××–Flag–××–mNeonGreen(1-236)
CHMP2A-NS3mut-green	CHMP2A(1-222)–××××–**GSMGSGSQHL**–××–NS3/4A(1-197)–××–Flag–××–mNeonGreen(1-236)
CHMP3 -NS3mut-green	CHMP3 (1-222)–××××–**GSMGSGSQHL**–××–NS3/4A(1-197)–××–Flag–××–mNeonGreen(1-236)
CHMP4B-NS3-green	CHMP4B(1-224)–××××–**GSMGSGSQHL**–××–NS3/4A(1-197)–××–Flag–××–mNeonGreen(1-236)

xxxx represents linker amino acids.
